# Short‐ and Long‐Term Healthcare Costs Attributable to Hepatitis B Among Newcomers to Ontario, Canada: A Matched Cohort Study

**DOI:** 10.1155/cjgh/2330427

**Published:** 2026-02-02

**Authors:** Kikanwa Anyiwe, Jordan J. Feld, Eleanor Pullenayegum, William W. L. Wong, Lena Nguyen, Beate Sander

**Affiliations:** ^1^ Institute of Health Policy, Management and Evaluation, University of Toronto, Toronto, Ontario, Canada, utoronto.ca; ^2^ Toronto Health Economics and Technology Assessment (THETA) Collaborative, Toronto General Hospital, University Health Network, Toronto, Ontario, Canada, uhn.ca; ^3^ Toronto Centre for Liver Disease, Toronto General Hospital, University Health Network, Toronto, Ontario, Canada, uhn.ca; ^4^ Sandra Rotman Centre for Global Health, University of Toronto, Toronto, Ontario, Canada, utoronto.ca; ^5^ Department of Medicine, Temerty Faculty of Medicine, University of Toronto, Toronto, Ontario, Canada, utoronto.ca; ^6^ Child Health Evaluative Sciences, The Hospital for Sick Children, Toronto, Ontario, Canada, sickkids.ca; ^7^ Dalla Lana School of Public Health, University of Toronto, Toronto, Ontario, Canada, utoronto.ca; ^8^ School of Pharmacy, University of Waterloo, Kitchener, Ontario, Canada, uwaterloo.ca; ^9^ ICES, Toronto, Ontario, Canada, ices.on.ca; ^10^ Public Health Ontario, Toronto, Ontario, Canada, publichealthontario.ca; ^11^ Health Systems and Policy Research Collaborative Centre, University Health Network, Toronto, Ontario, Canada, uhn.ca

**Keywords:** cost-of-illness, health economics, health expenditures, phase-of-care costing, viral hepatitis

## Abstract

**Background:**

Healthcare expenditures across the clinical trajectory of hepatitis B virus (HBV) infection are inadequately characterized, particularly among immigrants. Our aim is to quantify HBV‐attributable short‐ and long‐term costs among a population of newcomers to the province of Ontario.

**Methods:**

We performed a cost‐of‐illness study with an incidence‐based, matched cohort design, employing linked population‐based laboratory and health administrative data, and permanent residence data from Immigration, Refugees and Citizenship Canada (IRCC). We identified newcomers diagnosed with HBV between January 1, 2004, and December 31, 2018, and dichotomized the study cohort into individuals with or without liver complications before diagnosis. We used propensity score matching and phase‐of‐care costing to quantify monthly attributable costs for each of six HBV phases and longitudinal costs for one, five, and ten years following diagnosis. Costs were quantified in 2021 Canadian dollars.

**Results:**

Among *n* = 30,677 newcomers with HBV, 2.7 percent had complications before diagnosis. Mean monthly phase costs were higher for individuals with complications before diagnosis relative to those without for prediagnosis care ($439, 95% CI: $250–$645 vs. $22, 95% CI: $12–$34), initial care for HBV ($1545, 95% CI: $1196–$1945 vs. $331, 95% CI: $299–$369), continuing care for HBV ($537, 95% CI: $314–$760 vs. $73, 95% CI: $55–$92), and final care ($7271, 95% CI: $3749–$10,747 vs. $3430, 95% CI: $1813–$5061).

**Conclusions:**

Findings emphasize the dynamic nature of HBV‐attributable costs and highlight the importance of care following diagnosis and complication onset.

## 1. Introduction

Hepatitis B virus (HBV) infection presents a substantial health challenge; a recent study estimated a global HBV infection prevalence of 3.2%, representing HBV seropositivity for 257.5 million people [1]. Global regions of intermediate or high HBV endemicity (i.e., ≥ 2% HBV seroprevalence) include many low‐ and middle‐income countries (LMICs), as distinct from regions with low endemicity (i.e., < 2% seroprevalence) containing predominantly high‐income countries (HICs) [2–4]. The natural history of chronic HBV infection is complex, involving progression through multiple stages of infection defined by characteristic hallmarks of HBV serology, viral replication, and liver function that are associated with varying impacts on liver inflammation and injury [5]. Clinical care for chronic HBV requires ongoing serological, molecular, and biochemical monitoring along with liver surveillance to detect the onset of liver‐related complications, including cirrhosis, decompensated cirrhosis, hepatocellular carcinoma (HCC), liver failure, or liver‐related death [6, 7].

The complexity of the clinical progression of HBV is reflected in its impact on healthcare resources. HBV‐associated health expenditures are substantial and elevated in the presence of more severe liver disease and complications [8–12]. However, there is a paucity of research concerning HBV costs among immigrants, despite increased seropositivity in world regions with intermediate or high endemicity. Patterns in migration from LMICs to HICs are relevant when considering HBV population impacts. The elevated prevalence of HBV infection among people from HBV‐endemic regions highlights the importance of investigating costs among newcomers to low‐endemicity settings (i.e., immigrants), who experience a disproportionate HBV burden relative to non‐newcomers. Additionally, there is a lack of research concerning both short‐term (i.e., monthly) healthcare costs specific for clinically distinct events following HBV infection (e.g., diagnosis, onset of complications) and long‐term costs that extend longitudinally across the lifetime of individuals with HBV.

The purpose of this cost‐of‐illness study is to quantify the mean attributable short‐term and long‐term healthcare costs of HBV among newcomers to the province of Ontario.

## 2. Methods

We evaluated short‐ and long‐term HBV‐attributable costs using Ontario health administrative data, employing an incidence‐based, matched‐cohort study design. We followed guidelines of the REporting of studies Conducted using Observational Routinely collected health Data (RECORD) and the Strengthening the Reporting of Observational Studies in Epidemiology (STROBE) statements [13, 14].

### 2.1. Health Administrative Data

We used Public Health Ontario (PHO) laboratory HBV data, which includes results for the majority of HBV testing conducted for the province of Ontario, the most populous province in Canada (population ∼15.9 million). PHO data were linked to ICES health administrative data; these datasets were linked using unique encoded identifiers and analyzed at ICES. As a research organization, ICES houses health administrative datasets for the population of Ontario that is eligible for provincial healthcare coverage. ICES health administrative data were used to assess demographic characteristics and health‐relevant covariates and to estimate HBV‐attributable costs. The Immigration, Refugees and Citizenship Canada (IRCC) Permanent Residence Database was used to establish newcomer status.

### 2.2. Study Population

We assembled a cohort of individuals diagnosed with HBV between January 1, 2004, and December 31, 2018, using PHO laboratory data. We used a preexisting algorithm developed by Nanwa et al. [15] to define HBV diagnosis; this algorithm conforms to HBV definitions associated with the Centers for Disease Control and Prevention (CDC). Briefly, cohort inclusion (i.e., HBV infection) requires a positive test result for HBV surface antigen, HBV e antigen, HBV DNA, or HBV genotype (Appendix 1). We applied this algorithm to PHO serological and molecular tests performed between 2004 and 2014. Due to post‐2015 dataset changes regarding test result categories, we adapted an updated version of the algorithm for specimens obtained between 2015 and 2018. The date of HBV diagnosis corresponded to the date of the first positive test result. We created a study cohort of newcomers with HBV using IRCC data, by identifying newcomers as distinct from long‐term residents (Appendix 2), and defining newcomer status through the presence of an immigration landing date, beginning in 1985, within IRCC permanent residence data. Additional data sources are identified in Appendix 1.

We used a preestablished and validated set of algorithms developed by Lapointe‐Shaw et al. [16, 17] to identify the first occurrence of each of five liver complications: (i) cirrhosis, (ii) decompensated cirrhosis, (iii) HCC, (iv) liver transplant, and (v) liver‐related death. Additional algorithm details, including sensitivity and specificity values for cirrhosis, decompensated cirrhosis, and HCC algorithms, are presented in Appendix 1. The observation period for detecting complications extended from the earliest time data was available until the end of our follow‐up on December 31, 2020.

Clinical expertise and related literature [17] indicate that liver complications appearing much earlier than HBV diagnosis are likely unrelated to HBV infection. To determine a cutoff point for complications related to HBV for people with complications preceding HBV diagnosis, we performed joinpoint analysis on the time elapsed between the date of first complication onset and the date of HBV diagnosis. We identified an inflection point corresponding to 3 years prior to diagnosis. We categorized our study cohort into two groups using this 3‐year window: (i) No complications before diagnosis and (ii) Complications before diagnosis. The “No complications before diagnosis” group includes individuals with (a) no complications at all, (b) complications unrelated to their HBV diagnosis (i.e., their most recent complication occurred more than 3 years before diagnosis with HBV), and (c) complications after diagnosis (i.e., their first complication occurred after HBV diagnosis). The “Complications before diagnosis” group includes everyone else with a complication (i.e., their first complication occurred during the 3‐year window before diagnosis, including on the diagnosis date).

### 2.3. Matching

We used propensity score combined with hard matching to match newcomers with HBV (exposed) to comparators (unexposed), specified below, at three time points: (i) diagnosis date, (ii) complication onset date for individuals with complications after diagnosis, and (iii) 3 months before death for individuals who died between diagnosis and the end of observation (i.e., January 1, 2004, to December 31, 2020). We used logistic regression to calculate the propensity score—the probability of exposure (i.e., having HBV)—for each match, given relevant covariates, and performed one‐to‐one matching of exposed and unexposed individuals. Covariates for propensity score estimation included the following: age (5‐year categories), sex, income quintile, pregnancy, year of diagnosis, rurality, resource utilization band (Johns Hopkins ACG System Version 10; i.e., ACG System Resource Utilization Bands (RUBs) from 0 to 5), and marginalization indices [18] (i.e., residential instability, material deprivation, dependency, ethnic diversity). We conducted greedy nearest neighbor matching without replacement, using the logit of the propensity score, and a caliper width of 0.2 of the standard deviation of the logit of the propensity score [19].

For diagnosis date matching, we matched study cohort newcomers with HBV to newcomers without HBV from the general population (Appendix 2). Details are in Appendix 3. Following matching, 98.9% (Complications before diagnosis) and 93.4% (No complications before diagnosis) of individuals with HBV were successfully matched. For complication onset date matching, we performed a nested match—matching individuals who developed complications after diagnosis to those without any complications (i.e., relative to the 3‐year threshold)—to enable analysis of complications as a second exposure beyond HBV (Appendix 3). After matching, 74.3% of individuals with complications after diagnosis were matched. We additionally explored a less stringent matching algorithm for the nested analysis that resulted in an increased match rate of 90.4% (Appendix 4). For death date matching, newcomers with HBV who died during observation were matched to newcomers without HBV from the general population who died during the same period (Appendix 2); 100 percent of individuals (Complications before diagnosis and No complications before diagnosis groups) were matched (Appendix 3).

We examined standardized differences to evaluate the balance of covariates between exposed and unexposed groups. Standardized differences were ≤ 0.1 for all covariates.

### 2.4. Phase‐of‐Care Costing Approach

**Figure 1:**
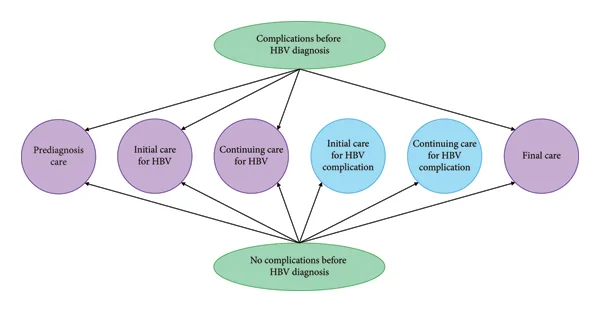
Allocation of observation time to phases of care. Available observation time was allocated to four possible phases for the “Complications before diagnosis” group (i.e., prediagnosis, initial care for HBV, continuing care for HBV, final care) and to six possible phases for the “No complications before diagnosis” group (i.e., prediagnosis, initial care for HBV, continuing care for HBV, initial care for HBV complication, continuing care for HBV complication, final care). The two complication‐specific phases were considered if complication onset occurred during follow‐up.

We used phase‐of‐care costing to quantify short‐term (i.e., phase‐specific) costs. We quantified mean monthly (i.e., 30‐day period) attributable costs for each of six HBV phases: (i) prediagnosis, (ii) initial care for HBV, (iii) continuing care for HBV, (iv) initial care for HBV complication, (v) continuing care for HBV complication, and (vi) final care. We determined the length of time spent in each phase using joinpoint analysis; we analyzed costs over time to identify trend inflection points. Phase lengths were confirmed by clinical insight and correspond to a previous HBV cost analysis [15] (Appendix 5). Available observation time was allocated to four phases for the Complications before diagnosis group (i.e., prediagnosis, initial care for HBV, continuing care for HBV, final care) and to six phases for the No complications before diagnosis group (i.e., prediagnosis, initial care for HBV, continuing care for HBV, initial care for HBV complication, continuing care for HBV complication, final care); the two complication‐specific phases were considered if complication onset occurred during follow‐up (Figure 1).

For the group with complications before diagnosis, observation time was allocated first to final care (duration of up to 3 months), then to initial care for HBV (up to 6 months); any time remaining between the initial care for HBV phase and either the final phase or the end of observation was then assigned to continuing care for HBV. The group with no complications before diagnosis included individuals with (i) no complications at all (i.e., relative to the 3‐year threshold) and (ii) complications after diagnosis. For those with no complications at all, observation time was allocated as above. For those with complications after diagnosis, available time was assigned first to final care (up to 3 months), then to initial care for HBV complication (up to 6 months), and finally to initial care for HBV (up to 6 months). Any time remaining between the initial care for HBV and the initial care for HBV complication phases was allocated to continuing care for HBV, and any intervening time between the initial care for HBV complication phase and either the final phase or the end of observation was assigned to continuing care for HBV complication. The prediagnosis phase was 9 months for individuals with and without complications before diagnosis. Phase lengths were established by exposed individuals and then applied to available observation time for matched unexposed comparators.

### 2.5. Phase and Long‐Term Costs

Costs were quantified using a costing macro available at ICES that derives individual‐level healthcare expenditures using health administrative databases [20]. HBV‐attributable phase costs were calculated as the mean net difference in standardized 30‐day costs between matched pairs for each phase. We used bootstrapping of the net cost difference between matched pairs to estimate 95% confidence intervals.

We calculated long‐term attributable costs adjusted for survival for one, five, and ten years following HBV diagnosis using an approach developed by Yabroff and others [21]. Briefly, we constructed life tables for individuals with HBV to calculate monthly probabilities of death, as well as a cumulative probability of survival at the end of each time period (i.e., one, five, or ten years). We additionally calculated monthly probabilities of postdiagnosis complications. We multiplied monthly (i.e., 30‐day) phase costs by the number of months spent in each phase and modified these cost estimates by either monthly probabilities of mortality for those who died or the cumulative probability of survival for individuals who lived. Total phase costs were weighted (i.e., multiplied) by the number of individuals dying each month or surviving at the end of the period. Adjusted long‐term costs were then quantified by calculating the total sum of costs across life table time intervals, dividing the total sum by the total number of individuals. Costs were quantified in 2021 Canadian dollars.

### 2.6. Stratified and Sensitivity Analyses

We conducted stratified analyses of phase costs. Prediagnosis, initial care for HBV, and continuing care for HBV phase costs were stratified by variables included in the hard match and measured at diagnosis (i.e., age, sex, income quintile, rurality, and resource utilization). Initial care for HBV complication and continuing care for HBV complication phase costs were stratified by complication onset hard match variables (i.e., age, sex, income quintile, rurality, and resource utilization). Final care costs were stratified by predeath hard match variables (i.e., sex and rurality). We also stratified phase costs by healthcare cost components, i.e., inpatient care, outpatient care, physician services, emergency visits, same‐day surgery, medications, laboratory tests, and other services (Appendix 6). Components are consistent with previous cost‐of‐illness analyses, including categories as aggregated in a study by Erman and others [22]. Cohort categorization was informed by a 3‐year complication onset threshold we identified through trend analysis. We additionally performed a sensitivity analysis of costs disregarding this 3‐year window and including all complications appearing before HBV diagnosis (Appendix 7).

Analyses were performed using SAS available through ICES (SAS Enterprise Guide Version 8.3). Joinpoint analyses were conducted with the Joinpoint Regression Program (Version 4.9.1.0) of the National Cancer Institute [23].

### 2.7. Research Ethics Approval

We obtained research ethics approval from the Research Ethics Boards of the University of Toronto and PHO. As our study involved analysis of health administrative data held at ICES, individual patient consent was not required. Research conforms to principles of the Declaration of Helsinki.

## 3. Results

### 3.1. Cohort Characteristics

For newcomers with HBV matched at diagnosis date (*n* = 30,677), baseline characteristics are presented in Table 1 for each of two complication groups: (i) complications before diagnosis (*n* = 841) and (ii) no complications before diagnosis (*n* = 29,836). Individuals with complications before diagnosis are older (mean age = 50.2 years) than individuals with no complications before diagnosis (mean age = 41.7 years), and individuals in both groups are more likely to be male (69.7% for individuals with complications before diagnosis and 56.1% for individuals with no complications before diagnosis). The distribution of individuals across income quintiles is similar for both groups and tends to be concentrated among lower quintiles. Regarding marginalization indices, people in both groups are concentrated in the lowest and highest residential instability quintiles, experience increased deprivation, and tend to live in areas with lower levels of dependency and higher levels of ethnic diversity. Individuals with complications before diagnosis demonstrate elevated resource utilization relative to individuals without complications before diagnosis. Residence in urban rather than rural geographic regions is almost universal for individuals with (100%) and without (99.9%) complications before diagnosis. A greater proportion of individuals with complications died during the observation window (28.7%) compared to individuals without complications before diagnosis (3.1%). The cohort had a mean follow‐up time of 3,277 days (SD = 1,381).

**Table 1:** Cohort characteristics for individuals with HBV matched at diagnosis.

	Overall	Complications before diagnosis	No complications before diagnosis
Total, *n*	30,677	841	29,836
Age, mean (SD)	41.89 (13.9)	50.15 (15.3)	41.66 (13.8)
Age group, *n* (%)			
≤ 19 years	658 (2.1)	12 (1.4)	646 (2.2)
20 to 34 years	9913 (32.3)	121 (14.4)	9792 (32.8)
35 to 49 years	11,378 (37.1)	275 (32.7)	11,103 (37.2)
50 to 64 years	6647 (21.7)	283 (33.7)	6364 (21.3)
≥ 65 years	2081 (6.8)	150 (17.8)	1931 (6.5)
Sex, *n* (%)			
Female	13,351 (43.5)	255 (30.3)	13,096 (43.9)
Male	17,326 (56.5)	586 (69.7)	16,740 (56.1)
Pregnant, *n* (%)			
No (of total *n*)	28,455 (92.8)	830 (98.7)	27,625 (92.6)
Yes	2222 (7.2)	11 (1.3)	2211 (7.4)
Year of diagnosis, *n* (%)			
2004	963 (3.1)	63 (7.5)	900 (3.0)
2005	984 (3.2)	60 (7.1)	924 (3.1)
2006	1203 (3.9)	68 (8.1)	1135 (3.8)
2007	1595 (5.2)	54 (6.4)	1541 (5.2)
2008	2107 (6.9)	53 (6.3)	2054 (6.9)
2009	3312 (10.8)	53 (6.3)	3259 (10.9)
2010	3536 (11.5)	46 (5.5)	3490 (11.7)
2011	2864 (9.3)	37 (4.4)	2827 (9.5)
2012	2817 (9.2)	142 (16.9)	2675 (9.0)
2013	2243 (7.3)	53 (6.3)	2190 (7.3)
2014	2180 (7.1)	52 (6.2)	2128 (7.1)
2015	1839 (6.0)	46 (5.5)	1793 (6.0)
2016	1806 (5.9)	36 (4.3)	1770 (5.9)
2017	1699 (5.5)	39 (4.6)	1660 (5.6)
2018	1529 (5.0)	39 (4.6)	1490 (5.0)
Income quintile, *n* (%)			
1 (lowest)	9583 (31.2)	292 (34.7)	9291 (31.1)
2	8404 (27.4)	208 (24.7)	8196 (27.5)
3	5533 (18.0)	150 (17.8)	5383 (18.0)
4	4409 (14.4)	107 (12.7)	4302 (14.4)
5 (highest)	2748 (9.0)	84 (10.0)	2664 (8.9)
Residential instability quintile, *n* (%)			
1 (lowest)	9665 (31.5)	210 (25.0)	9455 (31.7)
2	4434 (14.5)	135 (16.1)	4299 (14.4)
3	3535 (11.5)	101 (12.0)	3434 (11.5)
4	4460 (14.5)	127 (15.1)	4333 (14.5)
5 (highest)	8583 (28.0)	268 (31.9)	8315 (27.9)
Deprivation quintile, *n* (%)			
1 (lowest)	2855 (9.3)	79 (9.4)	2776 (9.3)
2	3495 (11.4)	94 (11.2)	3401 (11.4)
3	4882 (15.9)	150 (17.8)	4732 (15.9)
4	7970 (26.0)	199 (23.7)	7771 (26.0)
5 (highest)	11,475 (37.4)	319 (37.9)	11,156 (37.4)
Dependency quintile, *n* (%)			
1 (lowest)	9661 (31.5)	260 (30.9)	9401 (31.5)
2	7626 (24.9)	216 (25.7)	7410 (24.8)
3	6008 (19.6)	160 (19.0)	5848 (19.6)
4	4327 (14.1)	120 (14.3)	4207 (14.1)
5 (highest)	3055 (10.0)	85 (10.1)	2970 (10.0)
Ethnic diversity quintile, *n* (%)			
1 (lowest)	219 (0.7)	9 (1.1)	210 (0.7)
2	517 (1.7)	22 (2.6)	495 (1.7)
3	1316 (4.3)	40 (4.8)	1276 (4.3)
4	5031 (16.4)	166 (19.7)	4865 (16.3)
5 (highest)	23,594 (76.9)	604 (71.8)	22,990 (77.1)
Resource utilization band, *n* (%)			
0	2694 (8.8)	7 (0.8)	2687 (9.0)
1	1102 (3.6)	7 (0.8)	1095 (3.7)
2	7086 (23.1)	50 (5.9)	7036 (23.6)
3	13,599 (44.3)	251 (29.8)	13,348 (44.7)
4	5169 (16.8)	308 (36.6)	4861 (16.3)
5	1027 (3.3)	218 (25.9)	809 (2.7)
Rurality, *n* (%)			
Urban	30,657 (99.9)	841 (100.0)	29,816 (99.9)
Rural	20 (0.1)	—	20 (0.1)
Mortality during study, *n* (%)			
No	29,511 (96.2)	600 (71.3)	28,911 (96.9)
Yes	1166 (3.8)	241 (28.7)	925 (3.1)

*Note:* “Complications before diagnosis” includes individuals who experienced the onset of a first complication during the 3‐year period before HBV diagnosis. “No complications before diagnosis” includes individuals who had (a) no complications at all, (b) complications unrelated to their HBV diagnosis (i.e., their most recent complication occurred more than 3 years before diagnosis with HBV), and (c) complications after diagnosis (i.e., their first complication occurred after HBV diagnosis). Distributions of characteristics prior to matching are not presented due to suppression of small cells (i.e., ≤ 5 observations).

Abbreviation: HBV, hepatitis B virus.

Distributions of characteristics were generally similar to the population of newcomers with HBV prior to matching (*n* = 32,811), with slightly higher proportions of pregnant individuals and people living in rural regions in the prematched cohort (distributions are not presented due to suppression of small cells, i.e., ≤ 5 observations).

### 3.2. Phase and Long‐Term Costs

Attributable costs for prediagnosis, initial care for HBV, continuing care for HBV, and final care phases are higher for individuals with complications before diagnosis, relative to those with no complications before diagnosis (Table 2). For individuals with complications before diagnosis, Table 2 presents monthly (i.e., 30‐day) costs for prediagnosis ($439, 95% CI: $250–$645), initial care for HBV ($1,545, 95% CI: $1,196–$1,945), continuing care for HBV ($537, 95% CI: $314–$760), and final care ($7,271, 95% CI: $3,749–$10,747) phases. For individuals with no complications before diagnosis, Table 2 presents monthly costs for prediagnosis ($22, 95% CI: $12–$34), initial care for HBV ($331, 95% CI: $299–$369), continuing care for HBV ($73, 95% CI: $55–$92), initial care for HBV complication ($1,213, 95% CI: $1,079–$1,367), continuing care for HBV complication ($427, 95% CI: $353–$508), and final care ($3,430, 95% CI: $1,813–$5,061) phases.

**Table 2:** Mean monthly (30‐day) phase costs attributable to HBV for six HBV phases^∗^.

	Complications before diagnosis		No complications before diagnosis	
	*n* matched pairs	HBV‐attributable phase costs (95% CI)	*n* matched pairs	HBV‐attributable phase costs (95% CI)
Prediagnosis	841	$439 ($250 to $645)	29,836	$22 ($12 to $34)
Initial care for HBV	767	$1545 ($1196 to $1945)	29,765	$331 ($299 to $369)
Continuing care for HBV	728	$537 ($314 to $760)	28,845	$73 ($55 to $92)
Initial care for HBV complication	N/A	N/A	2732	$1213 ($1079 to $1367)
Continuing care for HBV complication	N/A	N/A	2592	$427 ($353 to $508)
Final care	240	$7271 ($3749 to $10,747)	922	$3430 ($1813 to $5061)

*Note:* “Complications before diagnosis” includes individuals who experienced the onset of a first complication during the 3‐year period before HBV diagnosis. “No complications before diagnosis” includes individuals who had (a) no complications at all, (b) complications unrelated to their HBV diagnosis (i.e., their most recent complication occurred more than 3 years before diagnosis with HBV), and (c) complications after diagnosis (i.e., their first complication occurred after HBV diagnosis).

Abbreviation: HBV, hepatitis B virus.

^∗^Phase costs were calculated as the mean net difference in standardized 30‐day costs between matched pairs; 95% confidence intervals were estimated with bootstrapping.

Long‐term attributable costs are higher for individuals with complications before diagnosis, compared to those with no complications before diagnosis (Table 3). For individuals with complications before diagnosis, long‐term costs for one, five, and ten years following diagnosis are $9,177 (95% CI: $6,644–$11,932), $22,697 (95% CI: $14,308–$31,292), and $37,250 (95% CI: $22,707–$52,003), respectively. For those with no complications before diagnosis, long‐term costs for one, five, and ten years following diagnosis are $2,657 (95% CI: $2,330–$3,029), $7,318 (95% CI: $5,974–$8,790), and $13,391 (95% CI: $10,738–$16,284), respectively (Table 3).

**Table 3:** Mean longitudinal costs attributable to HBV for one, five, and ten years after diagnosis^∗^.

	Complications before diagnosis (95% CI)	No complications before diagnosis (95% CI)
One year (adjusted for survival)	$9177 ($6644 to $11,932)	$2657 ($2330 to $3029)
Five years (adjusted for survival)	$22,697 ($14,308 to $31,292)	$7318 ($5974 to $8790)
Ten years (adjusted for survival)	$37,250 ($22,707 to $52,003)	$13,391 ($10,738 to $16,284)

*Note:* “Complications before diagnosis” includes individuals who experienced the onset of a first complication during the 3‐year period before HBV diagnosis. “No complications before diagnosis” includes individuals who had (a) no complications at all, (b) complications unrelated to their HBV diagnosis (i.e., their most recent complication occurred more than 3 years before diagnosis with HBV), and (c) complications after diagnosis (i.e., their first complication occurred after HBV diagnosis).

Abbreviation: HBV, hepatitis B virus.

^∗^Longitudinal costs were quantified for one, five, and ten years after the index date of HBV diagnosis. Survival and mortality data for individuals with HBV were used to adjust estimates for survival.

### 3.3. Stratified and Sensitivity Analyses

#### 3.3.1. Prediagnosis, Initial Care for HBV, and Continuing Care for HBV

Prediagnosis, initial care for HBV, and continuing care for HBV‐stratified phase costs are presented in Table 4. Regarding age, prediagnosis, initial care for HBV, and continuing care for HBV phase costs tend to be higher for older age groups (i.e., 50–64 and ≥ 65 years old) among individuals with and without complications before diagnosis.

**Table 4:** Stratified mean monthly (30‐day) HBV‐attributable costs for prediagnosis, initial care for HBV, and continuing care for HBV phases^∗^.

	Complications before diagnosis						No complications before diagnosis					
	Prediagnosis		Initial care for HBV		Continuing care for HBV		Prediagnosis		Initial care for HBV		Continuing care for HBV	
	*n* pairs	Cost (95% CI)	*n* pairs	Cost (95% CI)	*n* pairs	Cost (95% CI)	*n* pairs	Cost (95% CI)	*n* pairs	Cost (95% CI)	*n* pairs	Cost (95% CI)
Age group												
≤ 19 years	12	$201 ($83 to $322)	12	$776 ($194 to $1682)	12	$186 ($21 to $467)	646	$44 ($12 to $90)	646	$182 ($98 to $310)	634	$75 ($16 to $135)
20 to 34 years	121	−$7 (−$375 to $227)	120	$924 ($400 to $1595)	118	$491 ($65 to $1180)	9792	−$5 (−$17 to $6)	9788	$152 ($112 to $195)	9610	$82 ($61 to $108)
35 to 49 years	275	$180 ($10 to $345)	259	$1103 ($651 to $1559)	251	$422 ($172 to $733)	11,103	$16 ($4 to $28)	11,094	$326 ($271 to $392)	10,763	$64 ($42 to $86)
50 to 64 years	283	$709 ($252 to $1158)	251	$2025 ($1287 to $2907)	234	$647 ($203 to $1067)	6364	$62 ($27 to $103)	6336	$468 ($385 to $554)	6077	$50 (−$20 to $109)
≥ 65 years	150	$784 ($219 to $1432)	125	$2169 ($1096 to $3439)	113	$649 (−$76 to $1370)	1931	$62 (−$13 to $138)	1901	$881 ($649 to $1119)	1761	$147 ($36 to $262)
Sex												
Female	255	$667 ($313 to $1080)	236	$1617 ($1002 to $2353)	227	$800 ($313 to $1345)	13,096	$11 (−$3 to $27)	13,068	$262 ($215 to $312)	12,799	$87 ($64 to $112)
Male	586	$340 ($129 to $563)	531	$1514 ($1087 to $1998)	501	$417 ($169 to $676)	16,740	$31 ($16 to $48)	16,697	$385 ($339 to $437)	16,046	$61 ($33 to $90)
Income quintile												
1 (lowest)	292	$402 ($72 to $749)	265	$1281 ($782 to $1838)	252	$499 ($88 to $946)	9291	$27 ($3 to $53)	9265	$366 ($299 to $436)	8970	$63 ($22 to $100)
2	208	$329 (−$83 to $769)	185	$1042 ($450 to $1650)	180	$568 ($109 to $1083)	8196	$22 ($2 to $42)	8177	$287 ($232 to $345)	7956	$74 ($38 to $111)
3	150	$859 ($432 to $1333)	139	$2102 ($1081 to $3439)	127	$696 ($191 to $1211)	5383	$22 ($4 to $40)	5367	$339 ($259 to $425)	5184	$100 ($54 to $153)
4	107	$109 (−$110 to $342)	99	$1645 ($961 to $2447)	98	$225 (−$85 to $522)	4302	$15 (−$8 to $41)	4297	$349 ($272 to $443)	4155	$64 ($24 to $104)
5 (highest)	84	$513 ($99 to $979)	79	$2509 ($1206 to $4162)	71	$737 ($217 to $1461)	2664	$18 (−$12 to $54)	2659	$300 ($193 to $426)	2580	$62 ($13 to $114)
Rurality												
Urban	841	$439 ($250 to $645)	767	$1545 ($1196 to $1945)	728	$537 ($314 to $760)	29,816	$22 ($12 to $33)	29,745	$331 ($299 to $366)	28,825	$73 ($54 to $92)
Rural	0	—	0	—	0	—	20	−$10 (−$116 to $61)	20	$187 ($71 to $346)	20	−$169 (−$627 to $121)
Resource utilization band												
0	7	$6 ($1 to $10)	6	$230 ($41 to $472)	6	$461 ($137 to $986)	2687	$3 ($2 to $3)	2684	$192 ($121 to $285)	2651	$141 ($113 to $174)
1	7	$10 (−$18 to $35)	7	$2458 ($197 to $6801)	7	$1953 ($39 to $5508)	1095	$13 ($10 to $15)	1093	$193 ($114 to $307)	1072	$42 ($21 to $66)
2	50	$81 ($48 to $122)	47	$1517 ($620 to $2851)	47	$370 ($153 to $648)	7036	$17 ($14 to $20)	7032	$157 ($122 to $201)	6895	$33 ($0 to $60)
3	251	$154 ($85 to $228)	234	$1215 ($853 to $1761)	228	$331 ($146 to $556)	13,348	$9 (−$1 to $18)	13,327	$280 ($231 to $325)	12,899	$48 ($29 to $66)
4	308	$59 (−$121 to $219)	286	$1169 ($718 to $1620)	273	$614 ($274 to $999)	4861	−$36 (−$65 to −$7)	4846	$488 ($379 to $611)	4625	$120 ($60 to $186)
5	218	$1414 ($678 to $2098)	187	$2551 ($1484 to $3790)	167	$680 (−$79 to $1338)	809	$722 ($404 to $1070)	783	$2472 ($1875 to $3099)	703	$385 (−$145 to $797)

*Note:* “Complications before diagnosis” includes individuals who experienced the onset of a first complication during the 3‐year period before HBV diagnosis. “No complications before diagnosis” includes individuals who had (a) no complications at all, (b) complications unrelated to their HBV diagnosis (i.e., their most recent complication occurred more than 3 years before diagnosis with HBV), and (c) complications after diagnosis (i.e., their first complication occurred after HBV diagnosis).

Abbreviation: HBV, hepatitis B virus.

^∗^Covariates measured at diagnosis.

For individuals with complications before diagnosis, prediagnosis, initial care for HBV, and continuing care for HBV costs are higher for female individuals than for male individuals. Among those with no complications before diagnosis, prediagnosis and initial care for HBV costs are higher for male individuals compared with female individuals, and continuing care for HBV costs are higher for female individuals. There are inconsistent cost trends across income quintiles. For the group with no complications before diagnosis, prediagnosis, initial care for HBV, and continuing care for HBV costs are higher for individuals in urban relative to rural settings.

Regarding resource utilization, there is a consistent elevation of prediagnosis, initial care for HBV, and continuing care for HBV costs for individuals within the highest resource utilization level (i.e., RUB5), with observed increases in initial care for HBV and continuing care for HBV costs among those with complications before diagnosis for the second‐lowest resource utilization level (i.e., RUB1).

#### 3.3.2. Initial Care for HBV Complication and Continuing Care for HBV Complication

Stratified initial care for HBV complication and continuing care for HBV complication phase costs are presented in Table 5. Complication‐specific phase costs demonstrate an increasing gradient across age categories; costs are lower for younger age groups and higher for older age groups. Regarding sex, initial care for HBV complication and continuing care for HBV complication costs are elevated for male compared with female individuals. When stratified by income quintile, there is no apparent trend for initial care for HBV complication costs. However, continuing care for HBV complication costs tend to be somewhat higher for lower income quintiles (i.e., income quintiles 1–3).

**Table 5:** Stratified mean monthly (30‐day) HBV‐attributable costs for initial care for HBV complication and continuing care for HBV complication phases^∗^.

	No complications before diagnosis			
	Initial care for HBV complication		Continuing care for HBV complication	
	*n* pairs	Cost (95% CI)	*n* pairs	Cost (95% CI)
Age group				
≤ 19 years	14	$325 ($109 to $658)	14	$21 (−$53 to $87)
20 to 34 years	548	$707 ($478 to $984)	532	$196 ($97 to $324)
35 to 49 years	1067	$952 ($786 to $1133)	1031	$343 ($244 to $466)
50 to 64 years	867	$1695 ($1400 to $2020)	812	$641 ($495 to $818)
≥ 65 years	236	$1846 ($1213 to $2506)	203	$623 ($297 to $970)
Sex				
Female	1039	$1078 ($860 to $1293)	985	$387 ($264 to $508)
Male	1693	$1295 ($1115 to $1478)	1607	$451 ($355 to $553)
Income quintile				
1 (lowest)	901	$1263 ($1053 to $1514)	848	$451 ($329 to $597)
2	666	$1185 ($922 to $1494)	632	$538 ($342 to $742)
3	510	$1113 ($842 to $1427)	487	$436 ($291 to $591)
4	409	$1316 ($855 to $1820)	388	$256 ($158 to $368)
5 (highest)	246	$1135 ($751 to $1595)	237	$301 ($135 to $506)
Rurality				
Urban	2732	$1213 ($1079 to $1367)	2592	$427 ($353 to $508)
Rural	0	—	0	—
Resource utilization band				
0	≤ 5	^∗∗^	≤ 5	^∗∗^
1	≤ 5	^∗∗^	≤ 5	^∗∗^
2	189–194	$667 ($470 to $889)	180–185	$206 ($109 to $319)
3	1028	$1005 ($840 to $1190)	979	$330 ($250 to $425)
4	1235	$997 ($803 to $1185)	1185	$288 ($210 to $375)
5	275	$3308 ($2502 to $4184)	243	$1653 ($1110 to $2257)

*Note:* “Complications before diagnosis” includes individuals who experienced the onset of a first complication during the 3‐year period before HBV diagnosis. “No complications before diagnosis” includes individuals who had (a) no complications at all, (b) complications unrelated to their HBV diagnosis (i.e., their most recent complication occurred more than 3 years before diagnosis with HBV), and (c) complications after diagnosis (i.e., their first complication occurred after HBV diagnosis).

Abbreviation: HBV, hepatitis B virus.

^∗^Covariates measured at complication onset.

^∗∗^Data suppressed due to small cells (i.e., ≤ 5 observations).

#### 3.3.3. Final Care

For individuals with and without complications before diagnosis, final care costs are lower for male relative to female individuals (Table 6).

**Table 6:** Stratified mean monthly (30‐day) HBV‐attributable costs for final care phase^∗^.

	Complications before diagnosis		No complications before diagnosis	
	Final care		Final care	
	*n* pairs	Cost (95% CI)	*n* pairs	Cost (95% CI)
Sex				
Female	70	$7334 ($1381 to $13,595)	329	$5617 ($2611 to $8762)
Male	170	$7245 ($2960 to $11,780)	593	$2217 ($232 to $4298)
Rurality				
Urban	240	$7271 ($3749 to $10,747)	917–922	$3408 ($1725 to $5101)
Rural	0	—	≤ 5	^∗∗^

*Note:* “Complications before diagnosis” includes individuals who experienced the onset of a first complication during the 3‐year period before HBV diagnosis. “No complications before diagnosis” includes individuals who had (a) no complications at all, (b) complications unrelated to their HBV diagnosis (i.e., their most recent complication occurred more than 3 years before diagnosis with HBV), and (c) complications after diagnosis (i.e., their first complication occurred after HBV diagnosis).

Abbreviation: HBV, hepatitis B virus.

^∗^Covariates measured 3 months before death.

^∗∗^Data suppressed due to small cells (i.e., ≤ 5 observations).

#### 3.3.4. Healthcare Cost Components

Regarding individuals with complications before diagnosis, inpatient care constitutes the most substantial cost component across all phases (Figure 2(a)). Across phases, the second‐largest cost categories include outpatient care, physician services, and medications; the third‐largest components include physician services and outpatient care.

Figure 2(a) Complications before diagnosis: Mean monthly (30‐day) HBV‐attributable phase costs, stratified by cost components. Costs attributable to HBV stratified by cost categories, for each of four phases of care (prediagnosis, initial care for HBV, continuing care for HBV, final care). (b) No complications before diagnosis: Mean monthly (30‐day) HBV‐attributable phase costs, stratified by cost components. Costs attributable to HBV stratified by cost categories, for each of six phases of care (prediagnosis, initial care for HBV, continuing care for HBV, initial care for HBV complication, continuing care for HBV complication, final care).

**Figure:**
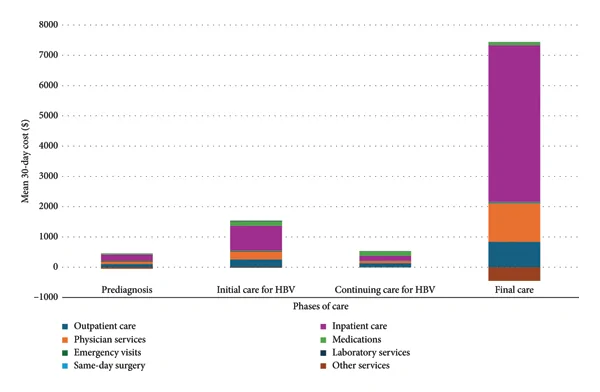
(a)

**Figure:**
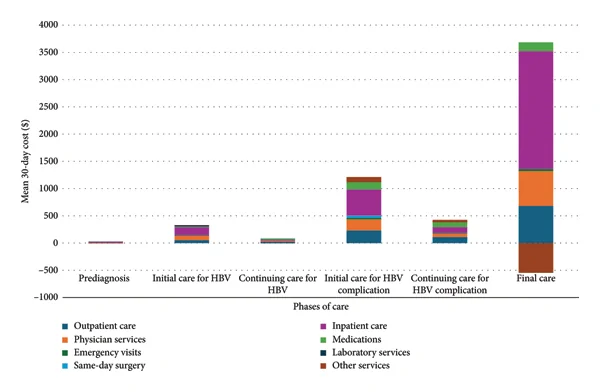
(b)

Regarding individuals with no complications before diagnosis, the highest costs are observed for either inpatient or outpatient care (Figure 2(b)). Outpatient care, inpatient care, laboratory services, physician services, and medications are represented among the second‐largest cost categories. The third‐most substantial components include physician services, outpatient care, inpatient care, and medications (Appendix 6).

#### 3.3.5. Sensitivity Analysis

When including all complications appearing before HBV diagnosis, phase and longitudinal costs are higher for individuals with complications before diagnosis (Appendix 7).

## 4. Discussion

We estimated HBV‐attributable phase‐specific and longitudinal healthcare costs for newcomers in Ontario. Higher costs per phase and long‐term costs for one, five, and ten years following HBV diagnosis were observed for individuals with complications before diagnosis, relative to those without. Increased costs were observed for older individuals with and without complications before diagnosis, and for female individuals with complications before diagnosis. Inpatient care, outpatient care, physician services, and medications represented the most substantial cost components.

Costs are elevated for initial care for HBV and final care phases relative to continuing care for HBV for individuals with and without complications before diagnosis. Additionally, where no complications occur before diagnosis, initial care for HBV complication costs are greater than continuing care for HBV and continuing care for HBV complication phases. Observed U‐shaped cost functions indicate that phase‐specific costs following HBV diagnosis are dynamic, rather than static. Peaks associated with initial care for HBV, initial care for HBV complication, and final care demonstrate that care services provided to individuals with HBV in periods following diagnosis, after complication onset, and preceding mortality are especially resource‐intensive. U‐shaped cost curves are consistent with existing phase‐of‐care cost analyses for chronic illnesses [21, 24–27]. In addition, longitudinal HBV costs over one, five, and ten years are considerable—consistent with an infection with chronic manifestation. Patterns in short‐term and lifetime costs align with a previous study calculating HBV‐attributable phase and long‐term costs for the general population of Ontario [15].

Our study is among the first to quantify phase and longitudinal costs attributable to HBV specifically among a population of newcomers or immigrants. Stratified costs highlight patterns within the HBV literature in other settings, including nonimmigrant populations. For example, older individuals with HBV generally display an increased occurrence of liver and nonliver comorbidities [28–33]. Differential costs for male and female individuals likely result from sex‐related variation in the likelihood of complications. Research indicates that men are more prone to the development and progression of complications following HBV infection, including liver fibrosis and HCC [34–37]. In our study, no clear trend is found regarding costs across resource utilization levels; however, elevated costs accompany the occurrence of liver complications. The literature identifies a positive association between growth in costs and increased comorbidities or disease severity [9, 10]. Among adults with chronic HBV, elevated inpatient and outpatient costs were observed for patients with an elevated comorbidity index or HCC [9]. Research investigating chronic HBV resource utilization and costs identified an association between an enhanced need for inpatient care and increased disease severity [10].

Across studies, costs vary markedly for different liver disease states and complications [8, 10–12, 38]. Patients with advanced liver disease with decompensated cirrhosis, HCC, or liver transplant demonstrate the most substantial resource use and costs [10]—a pattern common to several HBV cost analyses [8, 10–12, 38]. Moreover, studies reporting disaggregated health expenditures suggest that medications and hospitalizations constitute substantial cost components for care of HBV [8, 10, 11]. Literature‐identified results correspond with our findings, identifying inpatient care, outpatient care, physician services, and medications as the most sizeable HBV health expenditure categories.

Our study has several limitations. First, using a 3‐year window to identify complications before diagnosis may have excluded some HBV‐related liver complications. However, applying this 3‐year threshold increased the likelihood that complications were associated with HBV diagnosis as defined in our cohort; we additionally performed a sensitivity analysis including all complications. Second, using algorithm‐based complication onset to dichotomize our cohort into those with and without complications before diagnosis may unduly assign some individuals with preexisting complications to the second of these groups. For example, patients who presented with a liver complication and were tested for HBV during diagnostic workup for that condition may be mistakenly classified as being without a complication at diagnosis, if the HBV test result preceded confirmation of the complication. However, dichotomizing our cohort allows phase‐based exploration of costs associated with experiencing complications and continuing care for complications, which would otherwise not be feasible. Third, immigration data only extend backward to 1985; accordingly, there may be additional newcomers who were not included in our cohort due to the absence of an immigration landing date. Inclusion of individuals with post‐1985 landing dates introduces possible selection bias, as newcomers immigrating before this may demonstrate divergent trends in healthcare resource use and costs. As such, our results concerning attributable costs and patterns of expenditure may not be generalizable to the full population of newcomers with HBV. However, defining newcomers through the presence of a landing date established a more stringent criterion for study inclusion.

Fourth, as with many observational studies, there is differential follow‐up of individuals, depending on the time of diagnosis relative to the end of the study period; however, our study design and costing methods were developed to account for varying follow‐up across individuals. Fifth, the use of provincial health administrative data limits our analysis to populations (i.e., newcomers) eligible for universal publicly funded health coverage. Sixth, the ICES cost macro has incomplete cost availability windows for specific health administrative datasets pertaining to ambulatory care, primary care enrollment, mental health, and assistive device services. However, this limitation applies to both individuals with HBV and their comparators. Seventh, potential selection bias may have arisen from incomplete matching at complication onset, due to differences between matched and unmatched individuals in the distributions of certain characteristics. However, cost quantification performed for both main analysis complication onset matching and a less stringent comparator match yielded similar cost estimates for both the initial care for HBV complication and continuing care for HBV complication phases (Appendix 4). Eighth, bootstrapping yielded broad confidence intervals for particular care phases (e.g., final care), indicating uncertainty due to stochasticity in patient pathways and receipt of care. Such uncertainty may compromise the precision of quantified costs where there is substantial variability. However, bootstrapping permitted empiric estimation of confidence intervals based on observed data and allowed for intrinsic heterogeneity.

Our study also has several strengths. First, our incidence‐based phase‐of‐care costing design captured the dynamic nature of HBV‐attributable expenditures. Our approach enabled quantification of phase‐specific and lifetime costs through examination of how health spending changes following diagnosis, throughout care, and prior to death. Second, phase‐of‐care costing allowed allocation of any available observation time for individuals with HBV, despite time‐varied entries into and exits from the study cohort. Unlike other costing methods, phase‐based analysis permits the comprehensive use of differential follow‐up time. Third, we applied preestablished and previously validated definitions to assess the presence of key liver complications. Fourth, we identified people living with HBV through a multifactorial algorithm incorporating results for multiple serological and molecular HBV tests, to enhance the sensitivity of cohort inclusion. Fifth, matching procedures were carefully designed to consider important covariates and assess standardized differences to improve comparability of exposed and unexposed groups. Sixth, stratified cost analyses permitted a more comprehensive exploration of health expenditures for specific populations.

Our findings are germane to further research concerning the economic impact of HBV among immigrants in settings of low endemicity and are important for policymaking and priority‐setting regarding the provision of healthcare for individuals with HBV. Our analysis highlights the dynamic nature of HBV‐attributable costs and how health expenditures vary for demographic groups. Findings emphasize the significance of care following diagnosis and onset of liver complications and point to the policy importance of population‐level HBV surveillance to identify individuals who are seropositive for HBV infection, and to detect the development of liver complications.

## 5. Conclusions

Despite an increased burden of HBV among immigrants from endemic regions, there is a paucity of data within the literature regarding costs of HBV care specific for immigrants within countries of low HBV endemicity. Our study quantified population‐based phase‐specific and longitudinal HBV‐attributable costs for newcomers. Increased phase and long‐term costs were observed where complications occurred before diagnosis. Phase costs were observed to vary by age and sex. Publicly funded HBV screening and surveillance should be tailored for newcomer populations to address HBV‐attributable health expenditures. Future research should investigate the healthcare engagement of specific demographic groups among newcomers with HBV and the resulting impacts of engagement with care on liver health and well‐being.

## Ethics Statement

This project was approved by the Research Ethics Boards of the University of Toronto and Public Health Ontario. ICES is a prescribed entity under Ontario’s Personal Health Information Protection Act (PHIPA). Section 45 of PHIPA authorizes ICES to collect personal health information, without consent, for the purpose of analysis or compiling statistical information with respect to the management of, evaluation or monitoring of, the allocation of resources to, or planning for all or part of the health system. Projects that use data collected by ICES under Section 45 of PHIPA, and use no other data, are exempt from REB review. The use of the data in this project is authorized under Section 45 and approved by the Privacy and Legal Office at ICES.

## Disclosure

All authors revised and edited the manuscript and approved the manuscript in advance of submission. The funding sources had no role in the writing or publication of the manuscript. This study was presented as a poster at The Annual Canadian Liver Meeting 2025 of the Canadian Association for the Study of the Liver (CASL), the Canadian Network on Hepatitis C (CanHepC) and CanMASLD. An abstract was also published in the Canadian Liver Journal (Citation: The Annual Canadian Liver Meeting 2025 of the Canadian Association for the Study of the Liver (CASL), the Canadian Network on Hepatitis C (CanHepC) and CanMASLD. Can Liver J. 2025 Feb 25; 8 (1): 92–169). All authors revised and edited the manuscript and approved the manuscript in advance of submission.

## Conflicts of Interest

William W. L. Wong has received research support from the Canadian Liver Foundation and consulting fees from GSK. Jordan J. Feld has received research support from AbbVie, Bluejay, Gilead, GSK, and Vir and consulting fees from AbbVie, Arbutus, AstraZeneca, Gilead, Precision Biosciences, and Vir outside of the current work. The remaining authors declare no conflicts of interest.

## Author Contributions

All authors attest that they meet the ICMJE criteria for authorship. Kikanwa Anyiwe and Beate Sander conceptualized the study. Kikanwa Anyiwe, Jordan J. Feld, Eleanor Pullenayegum, William W. L. Wong, and Beate Sander contributed to design and methodology. Kikanwa Anyiwe and Lena Nguyen conducted data collection and curation. Kikanwa Anyiwe and Lena Nguyen performed data analyses. Kikanwa Anyiwe wrote the first draft of the manuscript.

## Funding

This study received funding from the Canada Research Chair (Tier 1) in Economics of Infectious Diseases held by Dr. Beate Sander [CRC‐2022‐00362] and was supported by an Ontario Graduate Scholarship award (Kikanwa Anyiwe).

## Supporting Information

Please refer to the Supporting information pdf for Appendices 1 to 7.

Appendix 1. Defining hepatitis B virus (HBV) infection, data sources, and liver complication algorithms. Table S1. Hepatitis B (HBV) laboratory tests. Appendix 2. Study cohort inclusion flowchart data. Table S2. Study cohort inclusion. Table S3a. Individuals with complications before HBV diagnosis who died during the study period. Table S3b. Individuals with no complications before HBV diagnosis who died during the study period. Table S4. Comparators from the general population. Table S5. Comparators from the general population who died during the study period. Appendix 3. Matching details. Appendix 4. Nested match on date of complication onset. Table S6. Comparison of nested matching approaches. Appendix 5. Phase length determination. Appendix 6. Phase costs stratified by cost components. Table S7. Complications before diagnosis: Mean monthly (30‐day) phase costs attributable to HBV, stratified by cost components. Table S8. No complications before diagnosis: Mean monthly (30‐day) phase costs attributable to HBV, stratified by cost components. Appendix 7. Sensitivity analysis. Table S9. Mean monthly (30‐day) phase costs attributable to HBV for six HBV phases (sensitivity analysis). Table S10. Mean longitudinal costs attributable to HBV for one, five, and ten years after diagnosis (sensitivity analysis).

## Supporting information


**Supporting Information** Additional supporting information can be found online in the Supporting Information section.

## Data Availability

The dataset from this study is held securely in coded form at ICES. While legal data‐sharing agreements between ICES and data providers (e.g., healthcare organizations and government) prohibit ICES from making the dataset publicly available, access may be granted to those who meet prespecified criteria for confidential access, available at https://www.ices.on.ca/DAS (email: das@ices.on.ca). The full dataset creation plan and underlying analytic code are available from the authors upon request, understanding that the computer programs may rely upon coding templates or macros that are unique to ICES and are therefore either inaccessible or may require modification.
