# Supplementary material for: Short‐ and Long‐Term Healthcare Costs Attributable to Hepatitis B Among Newcomers to Ontario, Canada: A Matched Cohort Study
Source: Can J Gastroenterol Hepatol. 2026 Feb 2;2026:2330427. doi: 10.1155/cjgh/2330427 (PMC13580811; doi:10.1155/cjgh/2330427)
Supplement: Supplementary file 1 [file CJGH-2026-2330427-s001.pdf]

Supplementary Appendices
Article: Short- and long-term healthcare costs attributable to hepatitis B among newcomers to Ontario, Canada: A matched cohort study

Canadian Journal of Gastroenterology and Hepatology

Authors:
Kikanwa Anyiwe[1,2], Jordan J. Feld[3,4,5], Eleanor Pullenayegum[6,7], William W. L. Wong[1,2,8,9], Lena Nguyen[9], Beate Sander[1,2,9,10]

Affiliations:
[1]Institute of Health Policy, Management and Evaluation, University of Toronto, Toronto, Ontario, Canada
[2]Toronto Health Economics and Technology Assessment (THETA) Collaborative, Toronto General Hospital, University Health Network, Toronto, Ontario, Canada
[3]Toronto Centre for Liver Disease, Toronto General Hospital, University Health Network, Toronto, Ontario, Canada
[4]Sandra Rotman Centre for Global Health, University of Toronto, Toronto, Ontario, Canada
[5]Department of Medicine, University of Toronto, Toronto, Ontario, Canada
[6]Child Health Evaluative Sciences, The Hospital for Sick Children, Toronto, Ontario, Canada
[7]Dalla Lana School of Public Health, University of Toronto, Toronto, Ontario, Canada
[8]School of Pharmacy, University of Waterloo, Waterloo, Ontario, Canada
[9]ICES, Toronto, Ontario, Canada
[10]Public Health Ontario, Toronto, Ontario, Canada

Corresponding author:
Kikanwa Anyiwe
k.anyiwe@mail.utoronto.ca

Appendix 1. Defining hepatitis B virus (HBV) infection, data sources, and liver complication algorithms
The algorithm used to identify individuals with HBV infection using Public Health Ontario (PHO) laboratory data was developed by Nanwa et al. in a previous study.[15] The presence of HBV infection was dependent on having a positive test for at least one of the following HBV assays: i) hepatitis B surface antigen (HBsAg), ii) hepatitis B e antigen (HBeAg), iii) hepatitis B DNA (HBV DNA), or iv) hepatitis B genotype. We additionally adapted this algorithm to incorporate post-2015 dataset changes.

Table S1. Hepatitis B (HBV) laboratory tests

**Test**	**Details**
Hepatitis B surface antigen (HBsAg)	Initial and confirmatory HBsAg testing was used to indicate HBV infection
Hepatitis B e antigen (HBeAg)	Required a positive test result to indicate HBV infection
Hepatitis B DNA (HBV DNA)	Required detection to indicate HBV infection
Hepatitis B genotype	Required detection to indicate HBV infection

Additional data sources included the following ICES-held datasets: Assistive Devices Program (ADP), Cancer Activity Level Reporting (ALR), Client Agency Program Enrolment (CAPE), Continuing Care Reporting System (CCRS), IRCC Permanent Residents database (CIC), Discharge Abstract Database (DAD), Home Care Database (HCD), Linked Delivering Mother and Newborns (MOMBABY), National Ambulatory Care Reporting System (NACRS), New Drug Funding Program (NDFP), National Rehabilitation Reporting System (NRS), Ontario Cancer Registry (OCR), Ontario Drug Benefit Claims (ODB), Ontario Home Care Administrative System (OHCAS), Ontario Health Insurance Plan Claims Database (OHIP), Ontario Mental Health Reporting System (OMHRS), Ontario Marginalization Index (ONMARG), Vital Statistics – Deaths (ORGD), Postal Code Conversion File (PCCF), Registered Persons Database (RPDB), Same Day Surgery Database (SDS)

Algorithms to identify cirrhosis, decompensated cirrhosis, and hepatocellular carcinoma (HCC) were developed and validated in a previous study by Lapointe-Shaw et al.[16] From the validation study, the algorithm we used for cirrhosis (i.e., ≥2 outpatient codes or ≥1 inpatient code for cirrhosis) had a sensitivity of 94 percent (95% CI: 92-95 percent), and a specificity of 85 percent (95% CI: 83-86 percent) among patients with HBV.[16] The algorithm we used for decompensated cirrhosis (i.e., ≥1 inpatient diagnostic code or ≥1 death code or ≥1 procedure code for decompensated cirrhosis) had a sensitivity of 88 percent (95% CI: 81-94 percent), and a specificity of 94 percent (95% CI: 93-95 percent) among patients with HBV.[16] The algorithm we used for HCC (i.e., ≥1 inpatient diagnostic code or ≥1 death code or ≥2 outpatient diagnostic codes or (≥1 outpatient cirrhosis code and ≥1 procedure code) or ≥1 Ontario Cancer Registry code) had a sensitivity of 97 percent (95% CI: 94-100 percent), and a specificity of 98 percent (95% CI: 98-99 percent) among patients with HBV.[16]

An algorithm to identify liver transplant was also developed by Lapointe-Shaw et al.[17] based on the validated previous algorithms for cirrhosis, decompensated cirrhosis, and HCC.[16,17] An algorithm for liver-related death was developed by the same research team, using death codes from the algorithms for decompensated cirrhosis, HCC, or liver transplant. While algorithms for liver transplant and liver-related death do not have directly-quantified sensitivity and specificity values, they are derived from successfully validated or pre-established algorithms.

Appendix 2. Study cohort inclusion flowchart data

We identified n=53,955 total individuals with HBV (i.e., both newcomers and long-term residents). After excluding individuals with missing data (i.e., missing covariates necessary for matching) and including only newcomers, there were n=32,811 newcomers with HBV who were eligible for matching to comparators from the general population. Of these, n=30,677 were successfully matched at diagnosis, and included within our study cohort of newcomers with HBV. Flowchart details are available in Table S2 below.

Table S2. Study cohort inclusion

**Number of individuals (n)**	**Inclusion / exclusion details**
53,955	Total individuals with HBV (i.e., newcomers and long-term residents)
53,180	After excluding individuals with missing data<br>• Missing encrypted identifier<br>• Invalid age (if age ≥ 111 years)<br>• Missing sex<br>• Missing date/year of diagnosis<br>• If death occurred before date of diagnosis<br>• Missing geographic location (i.e., urban or rural)<br>• Missing marginalization indices<br>• Missing resource utilization band<br>• Missing income quintile
32,811<br><br>850<br>31,961	Including newcomers only: i) Complications before diagnosis, ii) No complications before diagnosis)<br><br>Complications before diagnosis<br>No complications before diagnosis
30,677<br><br>841<br>29,836	Matched at diagnosis<br><br>Complications before diagnosis<br>No complications before diagnosis

Of n=841 individuals with complications before diagnosis, n=241 individuals died during the study period, and n=240 were matched at the final phase (i.e., three months before death). Of n=29,836 individuals with no complications before diagnosis, n=925 individuals died during the study period, and n=922 were matched at the final phase (i.e., three months before death). Flowchart details are available in Tables S3a and S3b below.

Table S3a. Individuals with complications before HBV diagnosis who died during the study period

**Number of individuals (n)**	**Inclusion / exclusion details**
841	Matched at diagnosis
241	Matched individuals who died during follow-up
240	After excluding individuals with missing data (i.e., covariates for final phase match measured three months before death)<br>• Missing sex<br>• Missing geographic location (i.e., urban or rural)
240	Matched at final phase (i.e., three months before death)

Table S3b. Individuals with no complications before HBV diagnosis who died during the study period

Number of individuals (n)	Inclusion / exclusion details
29,836	Matched at diagnosis
925	Matched individuals who died during follow-up
922	After excluding individuals with missing data (i.e., covariates for final phase match measured three months before death)<br>• Missing sex<br>• Missing geographic location (i.e., urban or rural)
922	Matched at final phase (i.e., three months before death)

Flowchart details regarding comparators from the general population are presented in Tables S4 and S5 below.

Table S4. Comparators from the general population

Number of individuals (n)	Inclusion / exclusion details
21,367,052	Sample of individuals from the general population eligible for publicly funded healthcare
16,531,276	After excluding individuals with missing data<br>• Missing birthdate (i.e., age)<br>• Invalid age (if age < 0 or age ≥ 111 years)<br>• Missing sex<br>• If death occurred before pseudo-date of diagnosis<br>• Missing geographic location (i.e., urban or rural)<br>• Missing marginalization indices<br>• Missing resource utilization band<br>• Missing income quintile
3,000,000	Random sample of three million individuals
584,451	Including newcomers only
584,231	Available for matching, after excluding individuals who died within three months of pseudo-date of diagnosis (i.e., length of final phase following pseudo date)

Table S5. Comparators from the general population who died during the study period

Number of individuals (n)	Inclusion / exclusion details
1,608,234	Sample of individuals from the general population eligible for publicly funded healthcare, who died during the study period
1,560,366	After excluding individuals with missing data (i.e., to be comparable to comparators in Table S4, and also to exclude those missing data for covariates in final phase match measured three months before death)<br>• Missing birth date (i.e., age)<br>• Invalid age (if age < 0 or age ≥ 111 years)<br>• If death occurred before date of final phase match (i.e., three months before death)<br>• Missing sex<br>• Missing geographic location (i.e., urban or rural)
1,000,000	Random sample of one million individuals
58,080	Including newcomers only
58,080	Available for matching, after excluding individuals who died within three months of the date of the final phase match

Appendix 3. Matching details

When matching at date of diagnosis, we matched newcomers with HBV from the study cohort to newcomers without HBV from the general population eligible for publicly funded healthcare (Appendix 2). A random pseudo-date of diagnosis was assigned to individuals without HBV, corresponding to the time period during which HBV diagnosis was observed (i.e., between 2004 and 2018) among the exposed individuals. The following covariates, measured on diagnosis index date, were used in propensity score estimation: age (5-year categories), sex, income quintile, pregnancy, year of diagnosis, rurality, resource utilization band (i.e., from 0 to 5, measured with the Johns Hopkins ACG System®, version 10), and marginalization indices (i.e., residential instability, material deprivation, dependency, ethnic diversity). Concurrent hard matching was done for the covariates age, sex, income quintile, pregnancy, year of diagnosis, rurality, and resource utilization band. Pregnancy was included as a covariate due to provincial HBV surveillance among pregnant individuals and increased pregnancy-associated healthcare costs. Following matching, 98.9 percent (Complications before diagnosis) and 93.4 percent (No complications before diagnosis) of individuals with HBV were successfully matched.

When matching at date of complication onset, for individuals without complications before HBV diagnosis, we performed a nested match within our study cohort of newcomers with HBV. We matched individuals who developed complications after diagnosis to those who did not display complications at all. A nested approach enabled analysis of complications as a second exposure beyond HBV, i.e., investigation of the incremental healthcare cost of this second exposure. A pseudo-date of onset was assigned, using random sampling of a Poisson distribution, to individuals for whom complications did not occur. Calculation of the propensity score was done using age, sex, income quintile, year of complication onset, rurality, resource utilization band, and marginalization indices (i.e., residential instability, material deprivation, dependency, ethnic diversity). Among these covariates, we hard matched on age, sex, income quintile, year of complication, rurality, and resource utilization band. After matching, 74.3 percent of individuals who developed complications after diagnosis were matched. We additionally explored a less stringent matching algorithm for the nested analysis that resulted in an increased match rate of 90.4 percent (Appendix 4).

When matching at death date, newcomers with HBV who died during the observation period were matched to newcomers without HBV from the general population who died during the same period (Appendix 2). We performed simultaneous propensity score and hard matching using the covariates sex, rurality, and year of mortality (i.e., three months prior to death). Other covariates were excluded from consideration to avoid obscuring analysis of pre-death costs by inappropriately matching on variables along the causal pathway of mortality resulting from HBV. Following matching, 100 percent of individuals (for Complications before diagnosis and No complications before diagnosis groups) were matched.

Appendix 4. Nested match on date of complication onset
We performed a nested match on the date of complication onset, to enable quantification of costs related to the onset of liver complications. The nested match involved individuals with no complications before HBV diagnosis; we matched a) individuals who experienced complications after diagnosis to b) individuals who did not demonstrate any complications during our follow-up period, applying a pseudo-date of complication onset to group b).

For the main analysis nested match, 74.3 percent of individuals were matched, while 25.7 percent remained unmatched. Comparing distributions of characteristics for matched and unmatched individuals, both groups had similar distributions for sex, rurality, dependency, ethnic diversity, and instability. Differences between the two groups were noted for age, income quintile, deprivation, resource utilization, and year of complication. Compared to matched individuals, unmatched individuals tended to be older, had a reduced proportion of people in the lower income quintiles and an increased proportion in the higher income quintiles, and were more likely to be categorized into the lowest deprivation quintile and less likely to be categorized into the highest deprivation quintile. Unmatched individuals were also more likely to fall into the highest resource utilization categories, and had increased proportions in the earliest years of complication onset, relative to years in the middle of observation time. Distributions are not presented due to suppression of small cells (i.e., ≤5 observations). However, quantification of costs following both the main analysis and less stringent nested matches reveal similar cost estimates for both the initial care for HBV complication and continuing care for HBV complication phases (Table S6).

We additionally explored a less stringent approach to matching, by removing income quintile from among the covariates on which individuals were hard matched during propensity score matching. A comparison of the two matching approaches – as well as mean monthly phase costs associated with each approach – is presented below. Phase costs for the two matching approaches are comparable, despite the difference in proportions of individuals matched.

Table S6. Comparison of nested matching approaches

	**Main analysis nested match**	**Less stringent nested match***
**Match covariates**	**Propensity score variables:**<br>-Age (5-year categories)<br>-Sex<br>-Income quintile<br>-Year of complication onset<br>-Rurality<br>-Resource utilization band<br>-Marginalization indices (Residential instability, Material deprivation, Dependency, Ethnic diversity)<br><br>**Hard match variables:**<br>-Age (5-year categories)<br>-Sex<br>-Income quintile<br>-Year of complication<br>-Rurality<br>-Resource utilization band	**Propensity score variables:**<br>-Age (5-year categories)<br>-Sex<br>-Income quintile<br>-Year of complication onset<br>-Rurality<br>-Resource utilization band<br>-Marginalization indices (Residential instability, Material deprivation, Dependency, Ethnic diversity)<br><br>**Hard match variables:**<br>-Age (5-year categories)<br>-Sex<br>-Year of complication<br>-Rurality<br>-Resource utilization band
**Match proportion**	74.3%	90.4%
**Mean monthly phase cost: Initial care for HBV complication**	$1,213<br>(95% CI: $1,079 to $1,367)	$1,189<br>(95% CI: $1,054 to $1,327)
**Mean monthly phase cost: Continuing care for HBV complication**	$427<br>(95% CI: $353 to $508)	$453<br>(95% CI: $378 to $535)

*Removing income quintile as a hard match variable

Appendix 5. Phase length determination

We performed an exploratory analysis of preliminary cost data of individuals with HBV to determine duration of phase lengths. We graphed preliminary monthly costs around the date of HBV diagnosis (i.e., two years before and two years after), and before mortality for individuals who died during follow-up (i.e., two years before death). We conducted joinpoint analysis of cost data to identify points of inflection indicating trend changes.[23] Graphing and joinpoint analyses were performed for an aggregate cohort of both newcomers and non-newcomers (i.e., long-term residents) with HBV, as well as for newcomers alone.

Phase lengths were additionally informed by clinical expertise. Maximum phase lengths – for pre-diagnosis (nine months), initial care for HBV (six months), initial care for HBV complication (six months), and final care (three months) phases – were consistent with a previous study quantifying HBV costs.[15]

Appendix 6. Phase costs stratified by cost components

We stratified phase costs by healthcare cost components – estimates are presented below. Differences between the sum of cost components and total phase costs are due to cost macro quantification methodology.

Table S7. Complications before diagnosis: Mean monthly (30-day) phase costs attributable to HBV, stratified by cost components

	Pre-diagnosis	Initial care for HBV	Continuing care for HBV	Final care
**Total***	$439 (95% CI: $250 to $645)	$1,545 (95% CI: $1,196 to $1,945)	$537 (95% CI: $314 to $760)	$7,271 (95% CI: $3,749 to $10,747)
**Outpatient care**	$106 (95% CI: $45 to $169)	$259 (95% CI: $179 to $351)	$134 (95% CI: $52 to $209)	$840 (95% CI: $580 to $1,122)
**Physician services**	$76 (95% CI: $53 to $101)	$257 (95% CI: $206 to $311)	$64 (95% CI: $38 to $91)	$1,277 (95% CI: $763 to $1,876)
**Emergency visits**	$26 (95% CI: $20 to $31)	$24 (95% CI: $17 to $32)	$6 (95% CI: $1 to $11)	$11 (95% CI: -$169 to $135)
**Same day surgery**	$0 (95% CI: -$7 to $6)	$13 (95% CI: $2 to $23)	$7 (95% CI: $2 to $13)	$31 (95% CI: -$6 to $79)
**Inpatient care**	$207 (95% CI: $92 to $319)	$815 (95% CI: $581 to $1,107)	$167 (95% CI: $50 to $301)	$5,179 (95% CI: $2,030 to $8,221)
**Medications**	$31 (95% CI: -$13 to $74)	$151 (95% CI: $79 to $225)	$143 (95% CI: $62 to $226)	$96 (95% CI: -$125 to $321)
**Laboratory services**	$5 (95% CI: $3 to $7)	$13 (95% CI: $10 to $16)	$2 (95% CI: $1 to $3)	$5 (95% CI: -$5 to $18)
**Other services**	-$47 (95% CI: -$117 to $21)	-$19 (95% CI: -$103 to $63)	-$1 (95% CI: -$65 to $64)	-$450 (95% CI: -$980 to $58)

*Cost components may not equal total, due to estimation procedures of cost macro

Table S8. No complications before diagnosis: Mean monthly (30-day) phase costs attributable to HBV, stratified by cost components

	Pre-diagnosis	Initial care for HBV	Continuing care for HBV	Initial care for HBV complication	Continuing care for HBV complication	Final care
**Total***	$22 (95% CI: $12 to $34)	$331 (95% CI: $299 to $369)	$73 (95% CI: $55 to $92)	$1,213 (95% CI: $1,079 to $1,367)	$427 (95% CI: $353 to $508)	$3,430 (95% CI: $1,813 to $5,061)
**Outpatient care**	$4 (95% CI: $1 to $8)	$55 (95% CI: $47 to $63)	$25 (95% CI: $20 to $31)	$232 (95% CI: $191 to $274)	$111 (95% CI: $82 to $144)	$683 (95% CI: $523 to $832)
**Physician services**	$4 (95% CI: $3 to $6)	$80 (95% CI: $73 to $86)	$14 (95% CI: $11 to $17)	$205 (95% CI: $183 to $229)	$62 (95% CI: $51 to $73)	$637 (95% CI: $389 to $929)
**Emergency visits**	$0 (95% CI: -$1 to $0)	$8 (95% CI: $5 to $11)	-$1 (95% CI: -$1 to $0)	$24 (95% CI: $19 to $30)	$9 (95% CI: $6 to $12)	$36 (95% CI: -$7 to $78)
**Same day surgery**	-$1 (95% CI: -$1 to $0)	$3 (95% CI: $2 to $4)	-$1 (95% CI: -$1 to $0)	$44 (95% CI: $38 to $51)	$5 (95% CI: $3 to $7)	$4 (95% CI: -$12 to $22)
**Inpatient care**	$11 (95% CI: $5 to $19)	$149 (95% CI: $127 to $176)	$16 (95% CI: $3 to $28)	$479 (95% CI: $398 to $578)	$107 (95% CI: $79 to $140)	$2,162 (95% CI: $647 to $3,622)
**Medications**	$0 (95% CI: -$3 to $2)	$14 (95% CI: $11 to $18)	$19 (95% CI: $15 to $22)	$130 (95% CI: $102 to $160)	$87 (95% CI: $70 to $105)	$162 (95% CI: $59 to $261)
**Laboratory services**	$6 (95% CI: $6 to $7)	$18 (95% CI: $17 to $19)	$4 (95% CI: $4 to $4)	$3 (95% CI: $1 to $4)	$1 (95% CI: $1 to $2)	-$1 (95% CI: -$5 to $2)
**Other services**	-$4 (95% CI: -$7 to -$1)	$3 (95% CI: -$1 to $7)	-$5 (95% CI: -$10 to $0)	$93 (95% CI: $62 to $131)	$43 (95% CI: $19 to $69)	-$545 (95% CI: -$870 to -$214)

*Cost components may not equal total, due to estimation procedures of cost macro

Appendix 7. Sensitivity analysis
Regarding our categorization of individuals into groups with and without complications before HBV diagnosis, we additionally conducted a sensitivity analysis disregarding the three-year window used to define complications relevant to HBV. Our sensitivity analysis included all liver complications occurring prior to diagnosis with HBV, for as far back as data was available.

Sensitivity analysis results – including mean monthly phase costs and longitudinal costs – are presented below.

Table S9. Mean monthly (30-day) phase costs attributable to HBV for six HBV phases (sensitivity analysis)*

	Complications before diagnosis		No complications before diagnosis	
	n matched pairs	HBV-attributable phase costs (95% CI)	n matched pairs	HBV-attributable phase costs (95% CI)
**Pre-diagnosis**	1,449	$330<br>(95% CI: $200 to $461)	29,228	$19<br>(95% CI: $9 to $31)
**Initial care for HBV**	1,373	$1,005<br>(95% CI: $790 to $1,225)	29,159	$331<br>(95% CI: $297 to $367)
**Continuing care for HBV**	1,332	$374<br>(95% CI: $243 to $525)	28,241	$70<br>(95% CI: $51 to $91)
**Initial care for HBV complication**	N/A	N/A	2,677	$1,079<br>(95% CI: $959 to $1,216)
**Continuing care for HBV complication**	N/A	N/A	2,546	$400<br>(95% CI: $325 to $483)
**Final care**	260	$7,070<br>(95% CI: $4,069 to $10,283)	902	$3,405<br>(95% CI: $1,695 to $4,968)

*Phase costs were calculated as the mean net difference in standardized 30-day costs between matched pairs; 95% confidence intervals were estimated with bootstrapping. HBV = hepatitis B virus.
'Complications before diagnosis' (in the sensitivity analysis) includes individuals who experienced onset of a first complication before HBV diagnosis. 'No complications before diagnosis' (in the sensitivity analysis) includes individuals who had a) no complications at all, and b) complications after diagnosis (i.e., their first complication occurred after HBV diagnosis).

Table S10. Mean longitudinal costs attributable to HBV for one, five, and ten years after diagnosis (sensitivity analysis)*

	Complications before diagnosis	No complications before diagnosis
**One year (adjusted for survival)**	$6,904<br>(95% CI: $5,171 to $8,764)	$2,618<br>(95% CI: $2,276 to $2,983)
**Five years (adjusted for survival)**	$19,388<br>(95% CI: $13,203 to $26,397)	$7,096<br>(95% CI: $5,674 to $8,632)
**Ten years (adjusted for survival)**	$33,414<br>(95% CI: $22,271 to $46,148)	$12,943<br>(95% CI: $10,128 to $15,990)

*Longitudinal costs were quantified for one, five, and ten years after the index date of HBV diagnosis. Survival and mortality data for individuals with HBV were used to adjust estimates for survival. HBV = hepatitis B virus.
'Complications before diagnosis' (in the sensitivity analysis) includes individuals who experienced onset of a first complication before HBV diagnosis. 'No complications before diagnosis' (in the sensitivity analysis) includes individuals who had a) no complications at all, and b) complications after diagnosis (i.e., their first complication occurred after HBV diagnosis).
